# Carbenoxolone does not cross the blood brain barrier: an HPLC study

**DOI:** 10.1186/1471-2202-7-3

**Published:** 2006-01-11

**Authors:** Yevgen Leshchenko, Sergei Likhodii, Wendy Yue, William M Burnham, Jose L Perez Velazquez

**Affiliations:** 1 Department of Pharmacology, University of Toronto, Toronto, Ontario, Canada; 2 Brain and Behaviour Programme and Division of Neurology, Hospital for Sick Children, Department of Paediatrics and Institute of Medical Science, University of Toronto, Toronto, Ontario, Canada

## Abstract

**Background:**

Carbenoxolone (CBX) is a widely used gap junctional blocker. Considering several reports indicating that transient gap junctional blockade could be a favourable intervention following injuries to central nervous tissue, and some current enthusiasm in studies using systemic injections of CBX, it is imperative to consider the penetration of CBX into central nervous tissue after systemic administrations. So far, only very indirect evidence suggests that CBX penetrates into the central nervous system after systemic administrations. We thus determined the amounts of CBX present in the blood and the cerebrospinal fluid of rats after intraperitoneal administration, using high performance liquid chromatography

**Results:**

CBX was found in the blood of the animals, up to 90 minutes post-injection. However, the cerebrospinal fluid concentration of CBX was negligible.

**Conclusion:**

Thus, we conclude that, most likely, CBX does not penetrate the blood brain barrier and therefore recommend careful consideration in the manner of administration, when a central effect is desired.

## Background

Carbenoxolone (CBX), a derivative of 18-glycyrrhetinic acid [1], is a widely used drug which, in addition to being a mineralocorticoid agonist and inhibiting 11-beta hydroxysteroid dehydrogenase [2], also blocks gap junctional communication [these issues nicely reviewed in reference [3]]. There has been an increasing enthusiasm in the use of CBX in studies where gap junctional coupling has to be altered, both in vitro [4-6] and in vivo [7-11]. Some of these in vivo studies attribute a possible effect of CBX in the central nervous system (CNS) of the animals [8,11] after systemic administration, thus assuming CBX crosses the blood brain barrier. However, the only study that suggested that CBX crosses the blood brain barrier is the Jellinck et al. determination of the inhibition of 11-beta-hydrysteroid dehydrogenase in brain after intraperitoneal (i.p.) injections [12].

To clarify this issue, we used a detection method based on high performance liquid chromatography (HPLC) [13], and assessed the drug concentration in blood and in cerebrospinal fluid (CSF) samples of adult rats injected intraperitoneally with CBX (50 mg/Kg). While CBX was found in the plasma, its concentration in CSF was insignificant.

## Results

**Figure 1:**
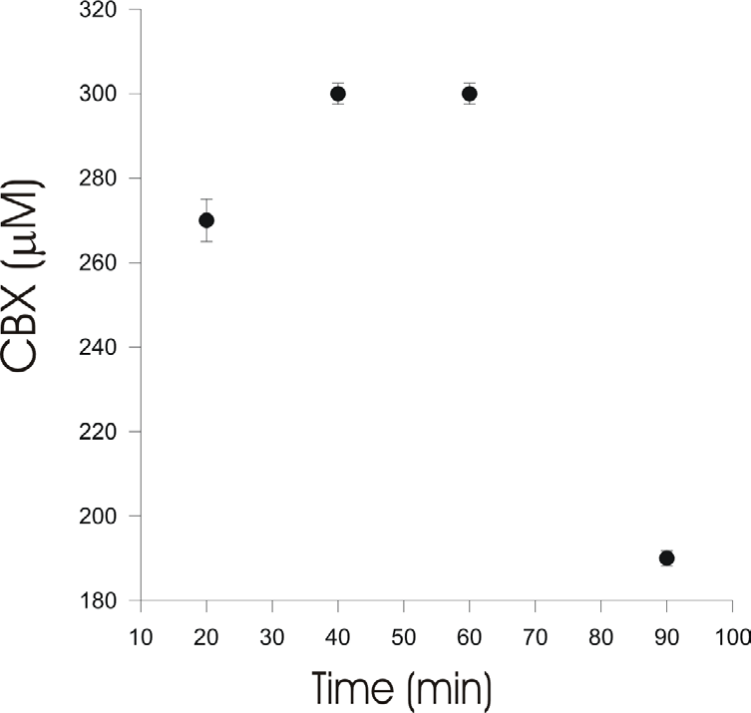
Time course of CBX detected in the plasma after an i.p injection of 50 mg/Kg. Maximal plasma concentration (mean +/- SD) was found between 40-and 70 minutes post-injection.

Following an i.p. injection of CBX (50 mg/Kg), blood samples were taken from the vein in the tail of the animal, and CSF samples collected from the cisterna magna. A total of 16 rats were used in this study. Figure 1 shows the HPLC-determined CBX concentration in the blood samples, at several time points. We note that, in plasma samples, we obtained about 15–20% of the theoretically estimated maximal CBX blood concentration (estimating a blood volume of 10% of body weight), that is, assuming all CBX was transferred to the circulation. However, one hour and a half post-injection, the concentration in blood had already dropped considerably to less than 200 μM. The concentration of CBX in CSF was negligible (<1 μM) at all time points tested, therefore we conclude that there is no transfer from the circulation to the CSF. In previous studies, we found that other compounds that are lipid soluble can be detected by our HPLC system at similar levels in plasma and in the CSF, such as acetone [14].

## Discussion

There is a current considerable interest in the possible roles of gap junctional communication in neuronal activity, and therefore a number of studies have used CBX as a gap junctional blocker and have attributed a central action of CBX after systemic administration. These studies include CBX effects on epileptiform activity [8,15] or on stereotypic behaviours [11]. Nevertheless, the only evidence that indicated, albeit indirectly, that CBX penetrates the blood brain barrier, was that presented in Jellinck et al. [12]; however, this evidence is very indirect because the studied relied on the CBX inhibition of 11-beta hydroxysteroid dehydrogenase, a relatively indirect measurement. As well, brain tissue was homogenized for these studies, which will include blood vessels and other tissues. A clearer determination of CBX presence in tissue samples can be obtained from specific fluid samples with the HPLC method developed by Zhang [13].

That CBX was not found in CSF samples should not really be surprising, as this molecule is polar and relatively large [1,3], and therefore we can conceive that it will be difficult to traverse the blood brain barrier. Hence, when looking for a central effect of CBX, a direct, intracranial administration will be more appropriate, using cannulae for example [9]. However, our determination of CBX in CSF samples still does not rule out completely the possibility of a very transient crossing of the blood brain barrier by the drug, if for instance, we envisage a situation in which CBX is concentrated in brain tissue with little spill over into the CSF. Because of the problems associated with determining drug presence in brain tissue, which would involve homogenisation of the tissue and thus bringing along arteries and CSF vessels, we did not attempt these analyses.

Considering current evidence that gap junctional blockers may have a beneficial CNS effect after traumatic injuries [5] or ischaemic insults [16,17], the issue of drug delivery could become of importance for possible therapeutic approaches. Hence, the creation of gap junctional ligands that traverse the blood brain barrier could be an important development. However, because of the breakdown of the blood brain barrier that occurs after traumatic/ischaemic, epileptic, or inflammation injury, a penetration into brain tissue can be expected in these conditions, consideration to be taken into account for possible therapeutic approaches.

## Conclusion

Our results suggest that CBX does not penetrate the blood brain barrier after i.p. administration, even though the possibility of a transient residence into brain tissue cannot be ruled out by these analyses. Hence, intracerebral injections are strongly recommended if the study is concerned with possible brain effects of the drug. In addition, intracerebral administration circumvents the potential confounding factor of systemic effects, which may be mediated by mineralocorticoid receptor activation and could contribute to the observed results.

## Methods

### Injections of CBX into animals and fluid withdrawal

CBX was purchased from Sigma. Male Long Evans rats (50–60 days old) were injected intraperitoneally (i.p.) with a dose of 50 mg/Kg. Blood (0.2 ml) was taken from the rat's tail vein, and CSF samples (0.1 ml) were taken at several time points after the drug injection, by puncturing the cisterna magna using a syringe equipped with a 30 G needle. Rats were anesthetized with halothane for all these procedures. The experiments were approved by the Hospital for Sick Children Animal care Committee

### High Performance Liquid Chromatography (HPLC)

We used the Aiglent Hewlett Packard Model 1100 Series, High performance liquid chromatography (HPLC) system (Aiglent Technologies), equipped with a variable Wavelength ultraviolet-visible detector. We followed the HPLC method previously developed to determine carbenoxolone concentration [13], using acetonitrile as component A of the mobile phase, and potassium phosphate buffer solution (pH 7.0) as component B. The mobile phase had a flow rate of 1.0 ml/min with a proportion 12% to 88% for components A and B of the mobile phase, respectively. Detection was performed with the UV detector at 254 nm.

## Abbreviations

CBX: carbenoxolone

CSF: cerebrospinal fluid

HPLC: high performance liquid chromatography

i.p.: intraperitoneal
